# Gastrodin Suppresses the Amyloid *β*-Induced Increase of Spontaneous Discharge in the Entorhinal Cortex of Rats

**DOI:** 10.1155/2014/320937

**Published:** 2014-10-30

**Authors:** Peng-zhi Chen, Hui-hui Jiang, Bo Wen, Shuan-cheng Ren, Yang Chen, Wei-gang Ji, Bo Hu, Jun Zhang, Fenglian Xu, Zhi-ru Zhu

**Affiliations:** ^1^Department of Physiology, Third Military Medical University, Chongqing 400038, China; ^2^School of Acupuncture and Tuina, Chengdu University of Traditional Chinese Medicine, Chengdu 611130, China; ^3^Department of Chemistry, Third Military Medical University, Chongqing 400038, China; ^4^Department of Physiology and Pharmacology, The Hotchkiss Brain Institute, Cumming School of Medicine, University of Calgary, Calgary, Canada T2N 4N1

## Abstract

Accumulated soluble amyloid beta- (A*β*-) induced aberrant neuronal network activity may directly contribute to cognitive deficits, which are the most outstanding characteristics of Alzheimer's disease (AD). The entorhinal cortex (EC) is one of the earliest affected brain regions in AD. Impairments of EC neurons are responsible for the cognitive deficits in AD. However, little effort has been made to investigate the effects of soluble A*β* on the discharge properties of EC neurons *in vivo*. The present study was designed to examine the effects of soluble A*β*_1−42_ on the discharge properties of EC neurons, using *in vivo* extracellular single unit recordings. The protective effects of gastrodin (GAS) were also investigated against A*β*_1−42_-induced alterations in EC neuronal activities. The results showed that the spontaneous discharge of EC neurons was increased by local application of soluble A*β*_1−42_ and that GAS can effectively reverse A*β*_1−42_-induced facilitation of spontaneous discharge in a concentration-dependent manner. Moreover, whole-cell patch clamp results indicated that the protective function of GAS on abnormal hyperexcitability may be partially mediated by its inhibitory action on A*β*_1−42_-elicited inward currents in EC neurons. Our study suggested that GAS may provide neuroprotective effects on A*β*_1−42_-induced hyperactivity in EC neurons of rats.

## 1. Introduction

Alzheimer's disease (AD) is the most common but incurable neurodegenerative disorder and is known to contribute to dementia among the elderly. Amyloid beta (A*β*) peptide, derived from the amyloid precursor protein, is regarded as the pivotal toxicant of AD and causes a variety of neuropathological changes, such as synaptic loss, dysfunction of neuronal transmission, and neuronal death [1]. Recently, increasing evidence has suggested that A*β*-induced perturbation of neuronal network activity may be a major and early contributor to AD pathogenesis. Aberrant neuronal network activity is observed in AD patients and transgenic mouse models of AD, which is thought to induce dysfunction and memory deficits [2, 3].

The entorhinal-hippocampal network is a vital circuit in memory consolidation and recall [4], which is seriously affected during the progression of AD [3]. The entorhinal cortex (EC) has been proven to be one of the earliest brain regions impaired in AD [5]. Radiological studies have also provided evidence that early-stage AD patients exhibit structural and functional changes of the EC [6, 7]. Previous studies suggested that elevated levels of A*β*, especially A*β*_1–42_, which has been described as the most toxic variant of A*β*, altered the excitability of neurons in the rat hippocampus [8, 9]. High levels of A*β*_1–42_ promoted suprasynchronization between individual rat prefrontal cortical neurons by increasing their excitability [10]. In addition, it is reported that A*β*_1–42_ could disturb the patterns of spontaneous discharge in the hippocampal CA1 region of rats [11]. However, there is little related electrophysiological data concerning EC neurons in the context of high level of A*β*_1–42_.

**Figure 1:**
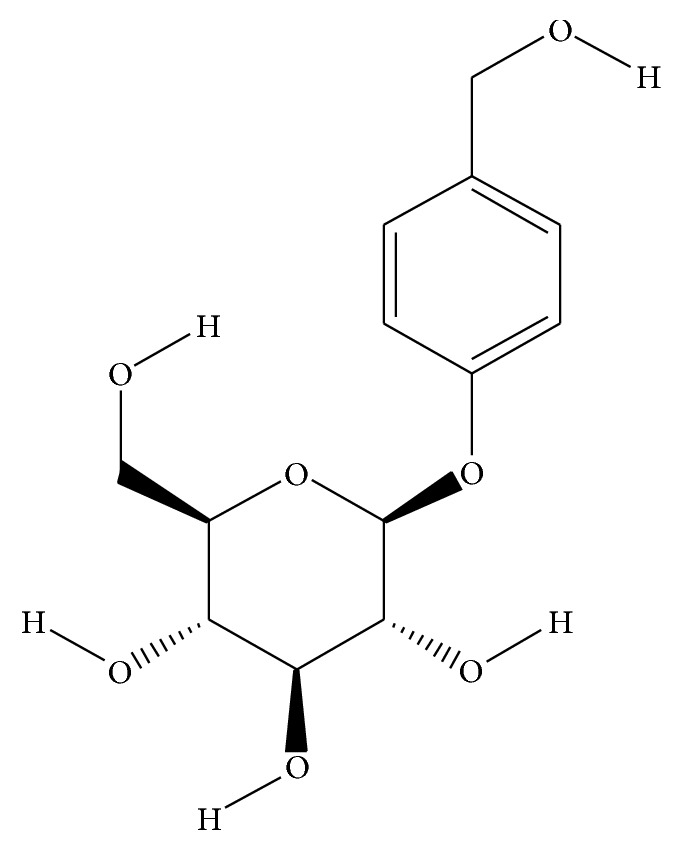
Structures of 4-hydroxybenzyl alcohol 4-O-beta-D-glucopyranoside (GAS).

Gastrodin (GAS) is a traditional Chinese medicine isolated from* Gastrodia elata* (Figure 1) and is regarded as one of the most important traditional medicines in Oriental countries. It is officially listed in the Chinese Pharmacopoeia and is used as an anticonvulsant, analgesic, and sedative against vertigo, general paralysis, epilepsy, and tetanus [12]. Modern clinical studies have demonstrated its efficiency as an antiepileptic drug [13] and its protective effects against cognitive decline in patients after cardiac surgery with cardiopulmonary bypass [14]. Furthermore, recent* in vitro* studies found that GAS had neuroprotective effects on the cellular model of AD induced by A*β*_25–35_ [15] and could facilitate learning and memory [16]. A recent study also demonstrated that A*β*_1–42_-related oxidative damage was decreased by GAS in primary cultured rat hippocampal neurons [17]. Despite these observations, whether GAS has neuroprotective effects against A*β*_1–42_-induced perturbation of neuronal network activity* in vivo* is unknown. Therefore, whether A*β*_1–42_ could disturb the spike discharge of EC neurons* in vivo* and whether the A*β*_1–42_-disturbed spike discharge could be remolded by GAS are significant questions. In the present study, we used extracellular recording and whole-cell patch clamp recording to investigate the effects of GAS on EC aberrant firing patterns induced by the local application of soluble A*β*_1–42_.

## 2. Materials and Methods

### 2.1. Animals and Surgery

Male Sprague-Dawley rats (200–250 g) were obtained from the Laboratory Animal Center at the Third Military Medical University in China. All protocols and procedures were approved by the University Animal Care and Use Committee. Animals were deeply anesthetized using urethane (1.5 g/kg) before being placed in a stereotaxic apparatus. The skull was exposed, and a small hole was drilled to expose EC region (from bregma: AP −6.6 mm and ML 4.7 mm; depth from skull surface: 6.8 mm) [18].

### 2.2. Drugs

All reagents were obtained from (Sigma-Aldrich, USA) with the exception of scrambled A*β*_1–42_ peptide (Anaspec, Fremont, CA, USA). Soluble A*β*_1–42_ was prepared as described previously [19]. In brief, A*β*_1–42_ was first dissolved in hexafluoro-2-propanol (HFIP) and aliquoted. HFIP was then removed by evaporation under vacuum, and the resulting clear peptide films were stored at −20°C. Prior to use, an aliquot of A*β*_1–42_ peptide film was dissolved in anhydrous dimethyl sulfoxide (DMSO) and added to ice-cold artificial cerebral spinal fluid (ACSF) to obtain a working concentration of 200 *μ*M. This solution was then incubated at 4°C for 24 h and centrifuged. The supernatant included the soluble A*β*_1–42_ preparation; the major species was A*β*_1–42_ monomer and also included the trimer, tetramer, and, to a lesser extent, the dimer [19]. Scrambled A*β*_1–42_ peptide was prepared in the same manner as A*β*_1–42_. GAS was dissolved in 0.9% sterile saline to deserved concentrations.

### 2.3. Single Unit Recordings

A five-barrel glass microelectrode (total tip diameter 3–10 *μ*m, resistance 5–20 MΩ) was used for electrophysiological recording and micropressure injection. The recording glass microelectrode was filled with a 0.9% sodium chloride solution. The other four barrels connected with a 4-channel pressure injector (PM2000B, Micro Data Instrument, Inc., USA) and were filled with different drugs as needed. The drugs were ejected on the surface of the firing cells with gas pressure [20]. The intrabarrel drugs' concentrations were chosen based on previous established works [8, 21, 22] and their efficacy to reliably alter neuronal firing. 0.5 *μ*L of drug was applied to the firing neurons during injection. The signals from the recording electrode were fed to an AM system amplifier (Carlsborg, WA, USA) and filtered with a band-pass of 0.3–10 kHz. A single unit was isolated and analyzed using Spike 2 (Cambridge Electronic Design Limited, London, UK). The signal was stored in a computer equipped with MATLAB analysis system for further offline analysis. The unit activities were subsequently analyzed per 500 s epoch for averaging discharge rates (bin width, 10 s), which were normalized relative to 500 s baseline values. To avoid the transient effects of the injection, windows of analysis were started 500 s after the injection of the drugs of interest.

### 2.4. Whole-Cell Patch Clamp Recordings

Sprague-Dawley rats (P12-21) were used in this study. After halothane anesthesia, each animal was decapitated and the brain was removed quickly. The brain was subsequently submerged in cold ACSF containing (in mM) 125 NaCl, 2.5 KCl, 25 NaHCO_3_, 1.25 KH_2_PO_4_, 1.2 MgSO_4_, 2 CaCl_2_, and 10 dextrose, bubbled with 95% O_2_-5% CO_2_, with a pH of 7.4. The brain was blocked, and an oscillating tissue slicer (Leica, VT1000, Wetzlar, Germany) was used to cut 400 *μ*m thick horizontal sections with a slicing angle of 20°. Slices were initially incubated for a minimum of 90 min at room temperature (22–24°C) in ACSF and were then transferred to and submerged in a recording chamber where they were perfused continuously with carbogen buffered ACSF at room temperature. Whole-cell patch clamp recordings were obtained from cell bodies of EC stellate neurons. Data acquisition was conducted with EPC10 amplifiers (HEKA Elektronik, Lambrecht/Pfalz, Germany). The signal was stored for offline analysis with Pulse/Pulse fit v.8.74 (HEKA Elektronik) and Igor Pro v.4.03 (WaveMatrics). Pipettes (4–8 MΩ) for whole-cell recordings were pulled on a horizontal micropipette puller (P-97, Sutter Instrument) from filamented capillary glass and were filled with a pipette solution containing (in mM) 145 K-gluconate, 0.5 EGTA, 2 MgCl_2_, 5 HEPES, 5 K-ATP, 0.4 Na-GTP, pH 7.4, and 290–295 mOsm. Liquid junction potential was calculated to be approximately −10 mV for the internal solutions, and membrane voltages were corrected offline. Series resistance was compensated by 80% and was continually monitored throughout the experiment. Neurons were discarded if the series resistance changed by more than 15%.

### 2.5. Data Analysis

Statistical analysis was made using statistical analysis software Origin 8.0 (Microcal, Inc, Northampton, MA, USA) and SPSS 18.0 (IBM, New York, NY). The values were presented as the mean ± S.E.M. Differences in the mean values among groups were analyzed using Student's *t*-test and one-way ANOVA. Values of *P* < 0.05 were considered significant.

## 3. Results

### 3.1. Soluble A*β*_1–42_ Increased the Spontaneous Firing Activities of EC Neurons

**Figure 2:**
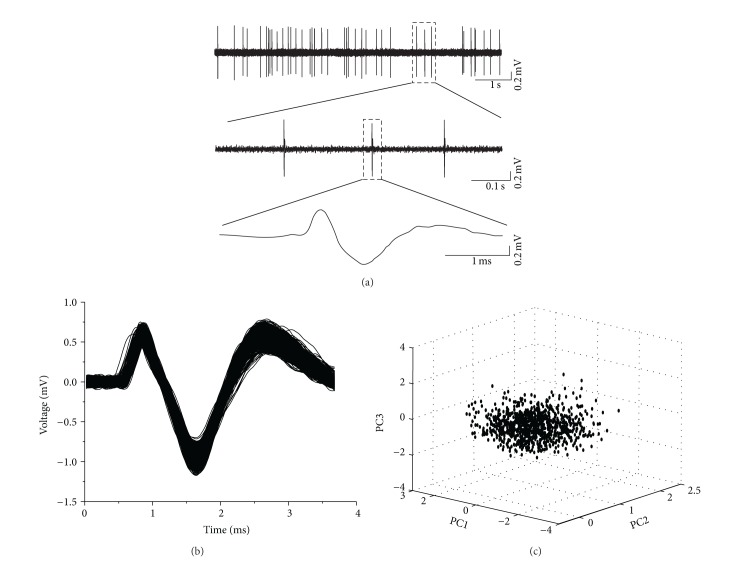
Recording and sorting of spontaneous discharge of EC neurons. (a) Single unit spontaneous discharge recorded in EC neurons (upper). The lower two panels are the enlargements of the indicated regions in the upper panel. (b) The overdrawn waveforms of firing events during a 30 min period form one cluster, indicating that all of the spikes were from one neuron. (c) Offline classification of spikes with principle component analysis (PCA). Every spike shown in (b) is projected onto the PCA-feature space shown in (c), further indicating that all of the spikes were from one neuron.

The changes in activity of EC neurons were determined by the spontaneous discharge before and after the application of soluble A*β*_1–42_.  Figure 2(a) shows a typical recording of unit activity from a single neuron. All of the spikes recorded in the present study showed a biphasic positive/negative waveform. A spike-sorting technique was used to separate single neuronal activity. Based on the different amplitudes and waveforms of spikes, recorded activities were sorted by principal components analysis (PCA).  Figure 2(b) shows representative sampling of waveforms from one EC neuron over 1800 s. Under basal conditions, the waveforms were stable, indicating that all of the recorded spikes were indeed from a single neuron.  Figure 2(c) presents offline classification of spikes with PCA. It is obvious that PCA analysis revealed one data cluster in the PCA-feature space, further indicating that all of the spikes recorded were from one neuron.

**Figure 3:**
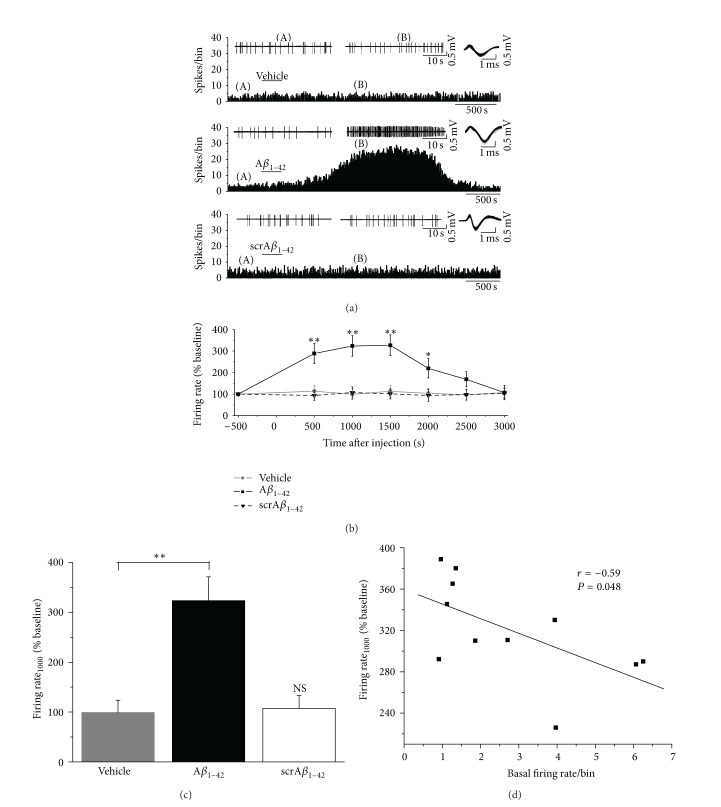
Effects of A*β*_1–42_ on the spontaneous firing of EC neurons. (a) Representative traces show the changes of firing activity of EC neurons after application of vehicle (upper), A*β*_1–42_ (middle), and scrambled A*β*_1–42_ (lower). Drugs were applied during the bar. The two traces in upper panel show firing patterns before (A) and after (B) the application of drugs. The upper right panel of each group displays the waveform of the recorded neuron across each protocol. No obvious change in the spike shape parameters was observed. (b) Time-response curves of the firing rate changes in EC neurons after administration of vehicle, A*β*_1–42_, and scrambled A*β*_1–42_ (^*^ *P* < 0.05, ^**^ *P* < 0.01 versus baseline values (500 s before injection)). (c) Bar graph summarizing effects of vehicle, A*β*_1–42_, and scrambled A*β*_1–42_ on the firing rate of EC neurons at 1000 s after local injection (^**^ *P* < 0.01, NS, no significant difference versus vehicle group). (d) Correlation between the firing rate at 1000 s after injection and the basal firing values in EC neurons. A*β*_1–42_ tended to enhance the low-rate spontaneous discharge more strongly.

Previous electrophysiological results elucidated that A*β*_1–42_ could excite hippocampal neurons* in vitro* [9]. In the following experiment, we further investigated the effects of soluble A*β*_1–42_ on the activities of EC neurons in anesthetized rats* in vivo*. Representative illustrations of firing changes in EC neurons after the application of vehicle or soluble A*β*_1–42_ are shown in Figure 3(a). The administration of 200 *μ*M A*β*_1–42_ increased the firing rates of EC neurons with an onset time about 300–900 s. As is shown in Figure 3(b), potentiation of the mean firing rate built up over the first 1000 s following the injection of A*β*_1–42_, stable for 500 s. Then, the mean firing rate gradually returned to baseline values, indicating that the effects of soluble A*β*_1–42_ on the discharge of EC neurons were reversible. At 1000 s after injection of A*β*_1–42_, the mean firing rate was 323.4 ± 47.9% of the baseline values (Figure 3(b), *n* = 11, *P* < 0.01 versus baseline (500 s before injection)). In contrast, the vehicle group, as a control, had no obvious effect on the firing activities of EC neurons (Figure 3(b), *n* = 11, *P* > 0.05 versus baseline). The mean firing rate of vehicle group at 1000 s after injection was 99.0 ± 24.5% of baseline, which was significantly different from that of the A*β*_1–42_ group (Figure 3(c), *n* = 11 for each group, *P* < 0.01). To ensure that the increase in discharge induced by A*β*_1–42_ was not due to nonspecific peptide effects, we applied a scrambled A*β*_1–42_ peptide to EC neurons. As shown in Figures 3(a)–3(c), the scrambled A*β*_1–42_ peptide did not have significant effect on the mean firing rate of EC neurons. The average firing rate of scrambled A*β*_1–42_ group at 1000 s after injection was 107.3 ± 26.0%, which was not significantly different from that of the vehicle group (Figure 3(c), *n* = 6 for scrambled A*β*_1–42_ group, *P* > 0.05). Altogether, these findings suggest that soluble A*β*_1–42_, but not its scrambled form, can significantly increase the spontaneous discharge of EC neurons.

As shown in Figure 3(d), we further analyzed the correlation between A*β*_1–42_-induced excitation at 1000 s after injection and the basal firing rate in the 11 neurons. Although modest, there was a negative correlation (*r* = −0.59, *P* < 0.05) between these two parameters, suggesting that the neurons with slower basal firing rate are more affected by A*β*_1–42_ in the EC.

### 3.2. GAS Prevented Soluble A*β*_1–42_-Induced Increase in Firing Activity

**Figure 4:**
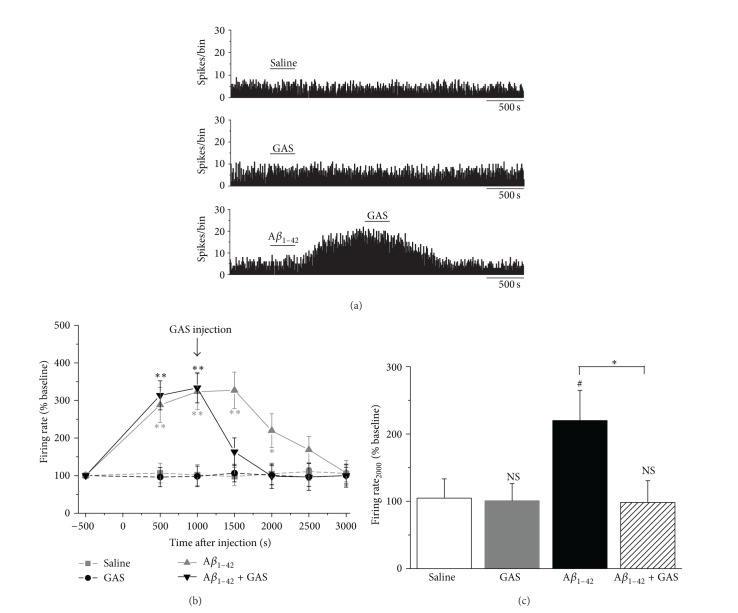
Inhibitory effects of GAS on A*β*_1–42_-induced increase in spontaneous discharge in EC region. (a) Representative traces of firing rate of EC neurons after the application of saline (upper), GAS alone (middle), or A*β*_1–42_ plus GAS (lower). Saline or GAS alone did not exert any obvious effect on the firing activities. Application of the GAS decreased the spontaneous discharge of A*β*_1–42_-induced hyperexcitation. (b) Time-response curves for saline, GAS alone, A*β*_1–42_ alone, and A*β*_1–42_ plus GAS groups on the firing rates of EC neurons (^*^ *P* < 0.05, ^**^ *P* < 0.01 versus baseline values). (c) Histograms showing the mean discharge rate of EC neurons in different groups at 2000 s after injection (NS, no significant difference versus vehicle group, ^#^ *P* < 0.05 versus saline group, ^*^ *P* < 0.05).

We then tested whether GAS can inhibit the soluble A*β*_1–42_-induced perturbation of spontaneous discharge. Both saline (used as a control) and 10 mM GAS alone had no obvious effect on the firing activities of EC neurons during the experiment (Figure 4(a)). The mean firing rates at 1000 s after application of the saline or 10 mM GAS were 101.7 ± 27.6% (*n* = 6, *P* > 0.05 versus baseline) and 98.1 ± 27.4% (*n* = 6, *P* > 0.05 versus baseline), respectively (Figures 4(b) and 4(c)). The administration of 200 *μ*M A*β*_1–42_ increased the spontaneous discharge of EC neurons (Figure 4(a)). At 1000 s after injection of A*β*_1–42_, the mean firing rate was 333.6 ± 39.9% of the baseline values (Figures 4(b) and 4(c), *n* = 10, *P* < 0.01 versus baseline). Applying 10 mM GAS to EC neurons significantly suppressed the A*β*_1–42_-induced increase in firing activity (Figure 4(a)). The firing rate at 2000 s after A*β*_1–42_ application (equal to that at 1000 s after GAS application) was 98.3 ± 32.3% of the baseline values, which was significantly less than that of A*β*_1–42_ alone group at the time point (Figures 4(b) and 4(c), 219.9 ± 44.9%, *n* = 11 for A*β*_1–42_ alone group, *P* < 0.05).

**Figure 5:**
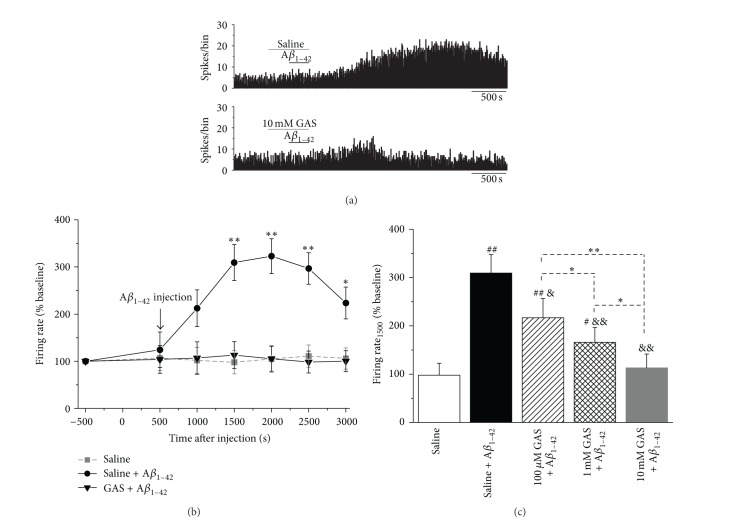
GAS pretreatment inhibited A*β*_1–42_-induced increase in firing rate of EC neurons in a concentration-dependent manner. (a) Representative traces show that pretreatment with 10 mM GAS (lower) prevented the A*β*_1–42_-induced increase in firing rate of EC neurons, whereas saline (upper) had no obvious effect. (b) Time-response curves of A*β*_1–42_-induced changes of firing rate in EC neurons, pretreated with saline or 10 mM GAS (^*^ *P* < 0.05, ^**^ *P* < 0.01 versus baseline values (500 s before saline or GAS injection)). (c) Concentration-dependent inhibitory effects of GAS on A*β*_1–42_-induced firing rate changes (^#^ *P* < 0.05, ^##^ *P* < 0.01 versus saline alone group, ^&^ *P* < 0.05, ^&&^ *P* < 0.01 versus saline plus A*β*_1–42_ group, ^*^ *P* < 0.05, ^**^ *P* < 0.01).

To further investigate the relationship between concentration and inhibitory effects of GAS, we pretreated the recorded EC neurons with different concentrations of GAS before A*β*_1–42_ injection. A representative illustration of the firing changes in EC neurons pretreated with GAS alone is shown in Figure 5(a). Saline or 10 mM GAS was applied when the spontaneous discharge was stable for 500 s. As shown in Figures 5(a) and 5(b), the administration of 200 *μ*M A*β*_1–42_ increased the firing rate of EC neurons pretreated with saline. At 1000 s after injection of A*β*_1–42_ (equal to that at 1500 s after saline application), the mean firing rate was 309.4 ± 38.3% of the baseline values (Figure 5(b), *n* = 6, *P* < 0.01 versus baseline). In contrast, following pretreatment with 10 mM GAS, no obvious increase in the firing activities in EC neurons was detected after the application of 200 *μ*M A*β*_1–42_ (Figures 5(a) and 5(b), 113.1 ± 28.4%, *n* = 6, *P* > 0.05 versus baseline). Analysis of the firing rates at 1000 s after application of A*β*_1–42_ (equal to that at 1500 s after pretreatment with different concentrations of GAS) revealed that the inhibitory effects of GAS against A*β*_1–42_ were concentration-dependent (Figure 5(c), saline alone group: 97.8 ± 24.5%, saline plus A*β*_1–42_ group: 309.4 ± 38.3%, 100 *μ*M GAS plus A*β*_1–42_ group: 216.9 ± 36.3%, 1 mM GAS plus A*β*_1–42_ group: 166.0 ± 30.5%, and 10 mM GAS plus A*β*_1–42_ group: 113.1 ± 28.4%, *n* = 6 for each group, ^#^ *P* < 0.05, ^##^ *P* < 0.01 versus saline alone group, ^&^ *P* < 0.05, ^&&^ *P* < 0.01 versus saline plus A*β*_1–42_ group, ^*^ *P* < 0.05, ^**^ *P* < 0.01). These results indicated that GAS had protective effects against the abnormal, A*β*_1–42_-induced activities of EC neurons.

### 3.3. GAS Blocked A*β*_1–42_-Elicited Inward Currents in EC Neurons

**Figure 6:**
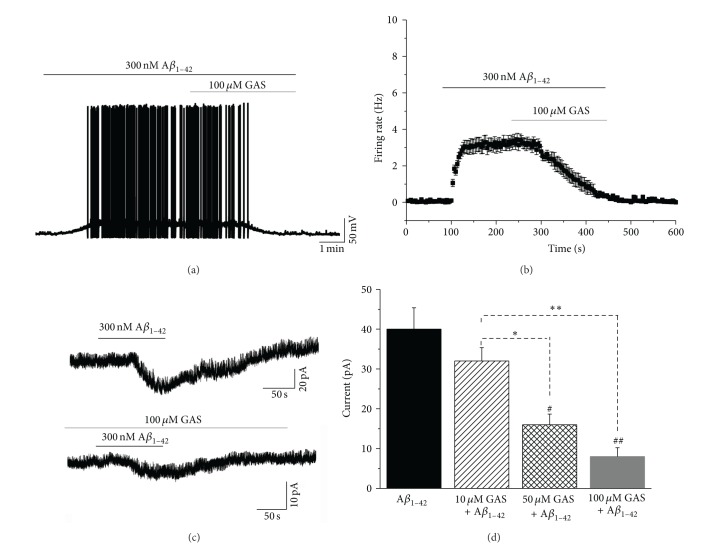
Inhibitory effects of GAS on A*β*_1–42_-elicited inward currents in EC neurons. (a) A*β*_1–42_-induced neuronal hyperexcitation was significantly inhibited in the presence of GAS. (b) Group data of 8 tested EC neurons showing the A*β*_1–42_-induced changes in the firing rate calculated at 5 s intervals. (c) 300 nM A*β*_1–42_ elicits inward currents in an EC neuron. GAS blocked the A*β*_1–42_-induced inward currents. (d) Group data of 6 tested EC neurons. The pretreatment of GAS decreased A*β*_1–42_-elicited inward currents in a dose-dependent manner (^#^ *P* < 0.05, ^##^ *P* < 0.01 versus A*β*_1–42_ alone group, ^*^ *P* < 0.05, ^**^ *P* < 0.01).

To explore the cellular mechanisms underlying the protective effects of GAS on A*β*_1–42_-induced perturbation of neuronal activity, we analyzed the effects of GAS on the excitability of soluble A*β*_1–42_-treated EC neurons in an* in vitro* slice preparation. As stellate neurons are the most abundant EC neurons [23], we took recordings from 8 randomly selected EC stellate neurons in current-clamp mode. Consistent with the* in vivo* results (Figure 3), bath application of 300 nM A*β*_1–42_ significantly increased the excitability of all recorded EC neurons (Figure 6(a)). Meanwhile, the robust A*β*_1–42_-induced firing of EC neurons was gradually diminished and completely abolished by the coapplication of 100 *μ*M GAS (Figures 6(a) and 6(b)). We next examined the effects of A*β*_1–42_ and GAS on whole-cell currents of EC neurons. In voltage-clamp mode, the recorded EC neurons were held at −70 mV. A brief (100 s) application of 300 nM A*β*_1–42_ elicited stable inward currents in the majority of the neurons (6/8). Intriguingly, administration of GAS significantly blocked the A*β*_1–42_-elicited inward currents in a dose-dependent manner (Figures 6(c) and 6(d)). The amplitude of A*β*_1–42_-elicited inward currents gradually decreased as the dose of GAS increased from 10 *μ*M to 100 *μ*M (Figure 6(d), A*β*_1–42_ alone group: 40.1 ± 5.4 pA, 10 *μ*M GAS plus A*β*_1–42_ group: 32.3 ± 3.4 pA, 50 *μ*M GAS plus A*β*_1–42_ group: 16.2 ± 2.6 pA, and 100 *μ*M GAS plus A*β*_1–42_ group: 8.0 ± 2.3 pA, *n* = 6 for each group, ^#^ *P* < 0.05, ^##^ *P* < 0.01 versus A*β*_1–42_ alone group, ^*^ *P* < 0.05, ^**^ *P* < 0.01). Altogether, these results suggest that GAS inhibits A*β*_1–42_-induced hyperactivity by blocking A*β*_1–42_-elicited inward currents in EC neurons.

## 4. Discussion

In the present study, we used extracellular single unit recording techniques to investigate the action of soluble A*β*_1–42_ on EC neuronal discharge* in vivo*. Our results demonstrated that local application of soluble A*β*_1–42_ significantly increased neuronal spontaneous discharge in the EC region (Figure 3). GAS prevented the alteration of the spontaneous discharge of EC neurons induced by A*β*_1–42_ (Figures 4 and 5). Whole-cell patch clamp results showed GAS blocked A*β*_1–42_-elicited inward currents of EC neurons (Figure 6).

### 4.1. GAS May Have Potential Therapeutic Value for A*β*-Induced Aberrant Activity

The EC occupies a central position in the limbic forebrain by providing bidirectional interconnections between the hippocampal formation and the rest of the cerebral cortex [4]. Animal experimentations and clinical observations in humans have demonstrated that the EC-hippocampal-neocortical circuit is fundamental in some forms of memory [4]. Some aspects of memory impairments in AD patients have been attributed to the damage of EC [24], which is one of the earliest and most severely damaged brain areas in this disease [5]. In the present* in vivo* study, we provided electrophysiological evidence that direct application of soluble A*β*_1–42_ could increase the spontaneous discharge of EC neurons (Figure 3). The production of A*β* and its secretion into the extracellular space are tightly regulated by neuronal activity. Increased neuronal activity enhances A*β* production, whereas blocking neuronal activity has the opposite effect [25]. We suspect that A*β*-induced hyperactivity of EC neurons can increase the production of A*β*, raising the possibility of a vicious cycle in which A*β* promotes its own production through alterations of EC neuronal activity. This vicious cycle may further impair the integrity and functions of neurons in the EC-hippocampal circuit, as A*β* synthesized by EC neurons can be transported via the perforant pathway to the hippocampus [3]. Thus, early interference in the EC to break this vicious cycle might be of therapeutic benefits, perhaps halting disease progression.

The ancient Chinese herb* Gastrodia elata* is considered to have several beneficial effects in treating headaches, dizziness, tetanus, and epilepsy [12, 13, 26, 27]. Importantly, GAS, the main active component of* Gastrodia elata*, can penetrate through the blood-brain barrier into the brain [13]. A recent study suggests that GAS protects primary cultured rat hippocampal neurons against A*β*-induced neurotoxicity, alleviating memory deficits and reducing neuropathology in a mouse model of AD [17]. These findings suggested that GAS might be effective in AD. The present study showed that administration of GAS reversed the A*β*_1–42_-induced spontaneous discharge alteration of EC neurons (Figure 4) and that this inhibitory effect of GAS was dose-dependent (Figure 5). In contrast, the same dose of GAS alone did not affect the firing activity of EC neurons (Figure 4). These results indicate that GAS may selectively act on spontaneous activity in abnormal A*β* states. We infer from the above results that GAS may be a beneficial agent in relieving the aberrant EC-hippocampal network activity in AD progression.

### 4.2. Potential Ionic Mechanisms Underlying the Protective Effects of GAS against A*β*-Induced Aberrant Activity

To explore the cellular mechanisms underlying the protective effects of GAS against A*β*_1–42_-induced aberrant activity, we used an* in vitro* slice preparation to perform whole-cell patch clamp recordings in EC neurons. Soluble A*β*_1–42_ caused a membrane depolarization and discharge in rat EC neurons (Figure 6(a)). These findings are consistent with the observation that direct application of soluble A*β*_1–42_ can induce EC neuronal hyperactivity* in vivo* (Figure 3). Furthermore, we found that A*β*_1–42_ elicited inward currents in EC neurons (Figure 6(b)). It has been reported that intracellularly or extracellularly applied A*β* or its fragments can modulate the function of ion channels, including potassium (K^+^), calcium (Ca^2+^), and sodium (Na^+^) channels [28]. In wild-type mice, A*β*_1–42_ decreases a suite of K^+^ conductance, including the delayed rectifier, the transient A-type, and the Ca^2+^-activated K^+^ currents [28].

In the present study, we found that the effects of A*β*_1–42_ were almost completely prevented by treatment with GAS (Figures 6(c) and 6(d)). A possible interpretation of the present findings is that GAS may exert its beneficial effects partially through the regulation of Na^+^ or K^+^ currents. Previous study has shown that GAS can dose-dependently reverse the pathologically altered Na^+^ and K^+^ currents in small dorsal root ganglion neurons in a model of diabetes [29]. In addition, GAS may also exert its beneficial effects through modulating Ca^2+^ currents. It has been shown that GAS can prevent glutamate-induced Ca^2+^ influx [30]. In contrast, A*β* enhances excitatory activity in glutamatergic synaptic networks and causes Ca^2+^ influx [31]. We infer that this opposing regulation of Ca^2+^ influx may contribute to the beneficial effects of GAS on the A*β*_1–42_-evoked abnormal excitability of EC neurons. It should be noted that ionic currents may not be the only target for GAS, and the effects of GAS on other cellular targets and signaling pathways cannot be excluded.

In summary, the current study showed that GAS treatment ameliorated A*β*_1–42_-induced perturbation of EC neuronal activity. These results suggested that GAS may be a potential candidate for AD therapy.
